# Elevated plasma levels of heparin-binding protein in intensive care unit patients with severe sepsis and septic shock

**DOI:** 10.1186/cc11353

**Published:** 2012-05-21

**Authors:** Adam Linder, Per Åkesson, Malin Inghammar, Carl-Johan Treutiger, Anna Linnér, Jonas Sundén-Cullberg

**Affiliations:** 1 Department of Clinical Sciences, Division of Infection Medicine, Klinikgatan 1, Lund University Hospital, SE-221 85 Lund, Sweden; 2 Department of Medicine, Center for Infectious Diseases F59, Karolinska Institute at Karolinska University Hospital Huddinge, SE-141 86 Stockholm, Sweden

## Abstract

**Introduction:**

Rapid detection of, and optimized treatment for, severe sepsis and septic shock is crucial for successful outcome. Heparin-binding protein (HBP), a potent inducer of increased vascular permeability, is a potentially useful biomarker for predicting outcome in patients with severe infections. Our aim was to study the systemic release and dynamics of HBP in the plasma of patients with severe sepsis and septic shock in the ICU.

**Methods:**

A prospective study was conducted of two patient cohorts treated in the ICU at Karolinska University Hospital Huddinge in Sweden. A total of 179 patients was included, of whom 151 had sepsis (126 with septic shock and 25 patients with severe sepsis) and 28 a non-septic critical condition. Blood samples were collected at five time points during six days after admission.

**Results:**

HBP levels were significantly higher in the sepsis group as compared to the control group. At admission to the ICU, a plasma HBP concentration of ≥15 ng/mL and/or a HBP (ng/mL)/white blood cell count (10^9^/L) ratio of >2 was found in 87.2% and 50.0% of critically ill patients with sepsis and non-septic illness, respectively. A lactate level of >2.5 mmol/L was detected in 64.9% and 56.0% of the same patient groups. Both in the sepsis group (n = 151) and in the whole group (n = 179), plasma HBP concentrations at admission and in the last measured sample within the 144 hour study period were significantly higher among 28-day non-survivors as compared to survivors and in the sepsis group, an elevated HBP-level at baseline was associated with an increased case-fatality rate at 28 days.

**Conclusions:**

Plasma HBP levels were significantly higher in patients with severe sepsis or septic shock compared to patients with a non-septic illness in the ICU. HBP was associated with severity of disease and an elevated HBP at admission was associated with an increased risk of death. HBP that rises over time may identify patients with a deteriorating prognosis. Thus, repeated HBP measurement in the ICU may help monitor treatment and predict outcome in patients with severe infections.

## Introduction

Sepsis is defined as the systemic inflammatory response to infection. In its more severe forms it causes tissue hypoperfusion, hypoxia, lactic acidosis, and organ dysfunction [1,2]. Despite increasing awareness of the diagnosis, faster administration of antibiotics and intravenous fluids, better technological support of organ function and other recent advances in treatment, the mortality rate in severe sepsis is approximately 35% [3-6]. Most severe infections are due to bacteria [7] and a large proportion (40%) of patients with severe sepsis has lung dysfunction associated with cardiovascular instability and deteriorating renal function [8]. Neutrophils have a pivotal role in the defense against bacterial infections [9] and in sepsis, the extravasation of neutrophils and plasma to the focus of infection is an important early step in the inflammatory process [10] initiating events that evolve into a systemic inflammatory response. Several inflammatory mediators, for instance, IL-6, lactate, and lipopolysaccharide binding protein (LBP), have been suggested as sensitive markers of disease progression in septic shock [11,12] and can also help differentiate bacterial from non-bacterial conditions [13].

Heparin-binding protein (HBP; also known as azurocidin and CAP37) is a multifunctional inflammatory mediator [14] with the ability to induce vascular leakage [15]. The protein is contained within the secretory and azurophilic granulae of human neutrophils [16] and is secreted upon stimulation of the leukocytic membrane-bound β2-integrins. HBP induces cytoskeletal rearrangement of endothelial cells, which leads to breakdown of cell barriers and an increase of the macromolecular efflux [15]. It has been shown that HBP is released upon neutrophil adhesion to endothelial cells, but also when neutrophils are activated by circulating protein complexes formed by streptococcal M protein and fibrinogen, a virulence mechanism which was shown to induce severe organ damage *in vivo *[17,18]. HBP was recently proposed as a biomarker for diagnosing bacterial meningitis [19] and for the early detection of patients in the emergency department (ED) at risk of developing severe sepsis and septic shock [20].

Considering the high morbidity and mortality associated with severe sepsis, a better risk-stratification of patients in the ICU may assist clinicians to manage the care of these patients more effectively and to improve outcome. Repeated lactate measurements have previously been studied as a marker of mortality and outcome [21]. Given the capacity of HBP to predict vascular leakage in ED patients, it is also an interesting candidate marker for monitoring patients with septic shock in the ICU. In the present study, serial plasma samples from critically ill patients treated in the ICU were analyzed. The potential use of lactate and HBP in diagnosing severe sepsis and septic shock, measuring treatment success, and predicting outcome, was evaluated.

## Materials and methods

### Study population

#### Patient selection

The present study consists of pooled results from two studies, with identical inclusion criteria, of the regulation of inflammatory markers in patients with severe sepsis and septic shock conducted between 2003 and 2009. Patients were recruited at Karolinska University Hospital Huddinge, a tertiary care hospital with 900 beds in Stockholm, Sweden. Study number one comprises 50 patients included between September 2003 and May 2005 [22]. Study number two has a total of 129 patients included between October 2005 and June 2009. The latter study also included a cohort of critically ill, but initially non-infected patients. Patients were evaluated daily on weekdays from 8 am to 4 pm by a trained research nurse and included on specified criteria (see definitions). After retrospective review of complete patient charts and laboratory and microbiological tests, one control patient was reclassified as septic shock and one as severe sepsis; two septic shock patients were reclassified as severe sepsis, and one as control. Finally, one patient was excluded from analysis altogether because a clear classification proved impossible. Due to hospital sub-specialization in Stockholm County, the Karolinska University hospital in Huddinge does not admit multi-trauma patients or those who have had elective lower GI, thoracic or brain surgery.

#### Ethical issues

The studies were conducted in accordance with the declaration of Helsinki and were approved by the Karolinska University ethics committee. Informed consent was obtained from the patients or next of kin.

#### Definitions

Sepsis, severe sepsis, and septic shock were defined according to previously described criteria [23]. The diagnosis of sepsis required a clinical assessment of infection together with a systemic inflammatory reaction syndrome (SIRS). Severe sepsis was defined as sepsis in addition to signs of acute reduction of organ perfusion as previously described [24]. Septic shock was defined as severe sepsis with hypotension requiring vasopressor support or mean arterial pressure <70 mm Hg for ≥30 minutes despite adequate fluid resuscitation. Control patients were defined as patients admitted for non-infectious critical illness and with an expected length of stay in the ICU longer than 24 hours.

#### Patient Characteristics

Individual clinical data were registered for the patients (Table 1). Severity of disease was measured by the Acute Physiology and Chronic Health Evaluation (APACHE) II [25] at admittance and also by daily Sepsis-related Organ Failure Assessment (SOFA) scores [26]. These and the final diagnoses were determined on the basis of complete patient charts and laboratory tests. The results of blood and other microbiological cultures were recorded.

**Table 1:** Characteristics of the study population.

Characteristics	Septic shock Number = 126	Severe sepsis Number = 25	Control group Number = 28	*P*-values
Gender - n (%) male	67 (53.2)	15 (60.0)	19 (67.9)	0.18
28 day mortality - n (%)	26 (20.6)	8 (32.0)	11 (39.3)	0.08
Final diagnosis - n (%)				
Lungs	30 (23.8)	13 (52.0)		
Intra-abdominal	55 (43.7)	3 (12.0)		
Urinary tract	17 (13.5)	4 (16.0)		
Skin/soft tissue	12 (9.5)	1 (4.0)		
Other septic conditions	12 (9.5)	4 (16.0)		
Cardiac arrest and/or myocardial infarction			15 (53.6)	
Liver transplantation (postop) and liver failure			6 (17.9)	
GI-bleeding			2 (7.1)	
Postoperative conditions			2 (7.1)	NA
Other non-septic conditions			3 (14.3)	NA
Comorbidities - n (%)				
Malignancy	51 (41.1)	8 (34.8)	4 (14.3)	0.03
Diabetes mellitus	4 (3.2)	1 (4.4)	3 (10.7)	0.22
Cardiac disease	18 (14.3)	4 (16.0)	7 (25.0)	0.38
Liver disease	0	0	6 (21.4)	<0.01
Other	43 (34.1)	9 (36.0)	6 (21.4)	0.40
Healthy	8 (6.4)	1 (4.0)	2 (7.1)	0.88
APACHE II score - mean	22.3	19.4	22.0	0.31
SOFA score - mean	10.5	8.3	9.3	0.01

APACHE, Acute Physiology and Chronic Health Evaluation; GI, gastrointestinal; n, number, SOFA, Sepsis-related Organ Failure Assessment.

#### Blood and culture samples

Blood and plasma samples were collected in 5 mL tubes at the time of inclusion or in the morning of the following day (0 hour) and after 24, 48, 96 and 144 hours. Ethylenediaminetetraacetic acid (EDTA) was used as the anticoagulant in plasma samples. Tubes were immediately centrifuged at 3000 rpm for ten minutes and separate aliquots of the plasma supernatants were stored at -80ºC until analysis. Routine biochemical analyses were performed. Two and three of the enrollment samples were not available for HBP and lactate analysis, respectively. Also, 25% of the samples from the 144-hour sampling point and half that many from the 96-hour time point were missing, mainly due to disrupted sampling after transfer of patients from the ICU to wards or other hospitals. Thus, samples from the latter part of the period represent a group with a slightly different composition than at baseline. It lacks those who have died, but also some who have improved.

### Laboratory analysis

The concentration of HBP was determined by ELISA as previously described [16]. Samples were coded and the researcher performing the analyses was blinded to patient data at the time. Briefly, microtiter plates (NUNC) were coated with a mouse monoclonal antibody directed against human HBP (2F23A) [27]. Patient plasma samples diluted 1/40 were added in duplicate. Each plate also contained calibration samples of known concentration of recombinant human HBP [28]. Plates were incubated with a polyclonal rabbit antiserum towards human HBP diluted 1/7000 [27]. The day-to-day variation of the assay had a coefficient of variance of <7%. Serum levels of IL-6 and IL-10 from the day of inclusion were determined by Luminex cytokine multiplex analyses using a Singleplex bead kit (Invitrogen, NY, USA) and the Luminex^100 ^instrument (Luminex, Austin, TX, USA). The minimum detectable level of IL-6 was <3 pg/ml and for IL-10 <5 pg/ml. The samples were analyzed in duplicate, using two different dilutions. Standard laboratory analyses were performed at the clinical chemistry laboratory, Karolinska University Hospital in Huddinge, according to the manufacturer´s instructions.

### Statistical analysis

Chi-square tests, one-way analyses of variance and Mann-Whitney U-tests were used to assess the distributions of covariates between the sepsis group and the control group. Spearman´s non-parametric correlation coefficient (rho) was used for calculating correlations between HBP and other investigated parameters in the patient groups. Cox proportional hazard models were used to estimate hazard ratios (HR) of 28-days mortality in sepsis patients according to initial and serial HBP-values modeled as categorical variable (<15 ng/mL, >15 ng/mL). Separate models were fitted with base-line values of HBP as predictor of death as well as base-line and change in HBP (HBP modeled as a time-dependent variable), adjusted for leukopenia (<4.0, >4.0), with s-creatinine and s-bilirubin, treated as continuous variables. Effect modification by degree of sepsis severity was assessed by examining the incidence rates of 28-day mortality in different strata of HBP and by entering an interaction term to the fully adjusted Cox models. Proportionalities of hazards were assessed graphically and by testing for a non-zero slope in a generalized linear regression of the scaled Schoenfeld residuals as a function of time.

The SPSS 14.0 software system (SPSS), GraphPad Prism 5 (Graph Pad Software, La Jolla) and STATA/SE (version 10.1 for Windows; StataCorp LP, College Station, TX) were used for calculations.

## Results

### Characteristics of the patients

A total of 179 patients were included in the study. Among these, 126 patients were diagnosed with septic shock and 25 patients with severe sepsis without shock. These 151 patients, with mainly abdominal and lower respiratory tract infections, were analyzed as a group, referred to as the sepsis group. The remaining 28 patients, referred to as the control group, were admitted due to non-infectious conditions (mainly myocardial infarction and cardiac arrest; see Table 1 for details).

In the sepsis group, only 6% were considered previously healthy. Blood cultures were obtained from 128/151 patients, of which 64 (50.0%) were positive; all sepsis patients received antibiotics. The mean APACHE II and SOFA scores at enrollment were 21.4 and 10.1, respectively; 73.5% were treated with steroids and 93% were treated with vasopressor drugs. Eighty percent had been treated with vasopressors for more than 12 hours before the first blood sample was taken.

In the control group, 14 patients (50.0%) developed an infection sometime during the days following inclusion and 24 patients (86%) were given antibiotics either as treatment, or as part of a prophylaxis regimen. Twenty-one control patients (75.0%) had shock at enrollment and 28-day mortality was 39%. The mean APACHE II score and SOFA score at enrollment was 22.1 and 9.3, respectively, and vasopressors were used in 20 patients (75.0%), as outlined in Table 1.

### Plasma levels of HBP, lactate, CRP, and WBC at enrollment

**Figure 1:**
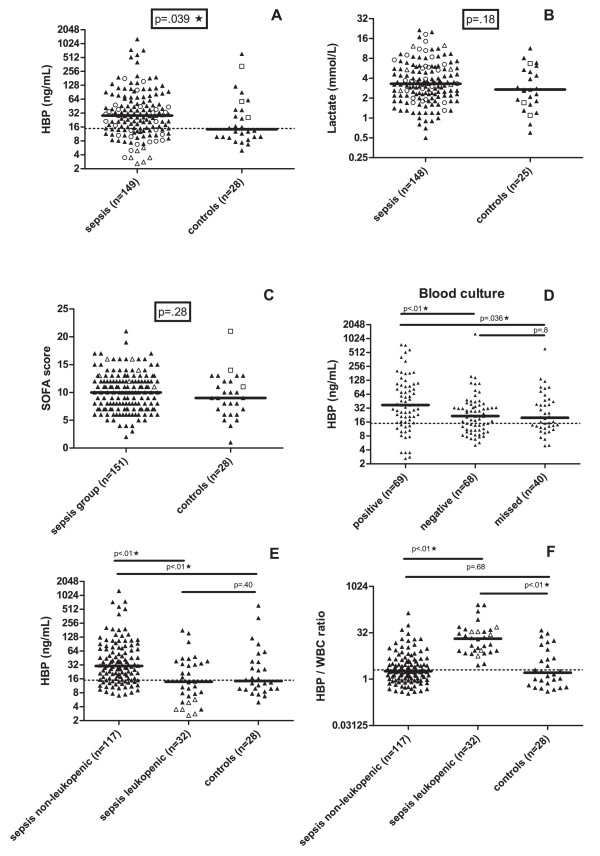
**Plasma levels of HBP and lactate, SOFA score at enrollment in the ICU, correlation with HBP and blood culture results, and HBP concentration and HBP/WBC ratio**. Each dot represents the concentration in an individual plasma sample at enrollment of HBP (**A**) and lactate (**B**). **C **shows SOFA scores at enrollment. **D **describes HBP concentration in patients in comparison with blood culture results. **E-F **shows patients divided into non-leukopenic, leukopenic and controls describing HBP concentration (E), and HBP/WBC ratio (F). The patient groups are described in the Methods section. Bars represent median values. The suggested cut-off value for HBP is marked at 15 ng/mL (A, D-E) and ratio value 2 (F). Mann-Whitney´s non-parametric test was used for comparisons between the groups and *P*-values are given. Star symbols indicate significant difference (*P *<.05). Open circles represent patients with transient leukopenia, open triangles represent patients with persistent leukopenia, and open squares control patients with a positive blood culture. CRP, C-reactive protein; HBP, heparin-binding protein; SOFA, Sepsis-related Organ Failure Assessment; WBC, white blood cells.

Enrollment samples were available for HBP and lactate analysis in 149 and 148 sepsis patients, respectively. A HBP concentration in plasma of ≥15 ng/mL was considered to be significantly elevated based on our previous report of plasma HBP levels in patients with severe sepsis [20]. At enrollment, HBP levels were significantly higher in both the septic shock group (*P *=.034) and in the combined sepsis group (median 28, range 3 to 1.277 ng/mL) (*P *= .029) compared to the control group (median 14.5 to 614 ng/mL) (Figure 1A), although there was no significant difference between the septic shock (median 30.3 to 1.277 ng/mL) and the severe sepsis group (median 24.7 to 107 ng/mL) (*P *=.25). Lactate levels were significantly elevated in the septic shock group compared to the severe sepsis group (*P *=.030), but no significant differences were found between the septic shock (*P *=.090) and the sepsis group (*P *=.55) compared to the control group (Figure 1B). C reactive protein (CRP), IL-6 and IL-10 levels were significantly higher (*P *<.01) but there was no significant difference in WBC levels (*P *=.49) or SOFA scores (*P *=.28) (Figure 1C) in the sepsis group compared to controls at enrollment. HBP levels were significantly higher among patients with positive blood cultures compared to those with negative blood cultures (*P *<.01) (Figure 1D). Of the patients with sepsis, 73.2% (109/149) had an HBP level exceeding 15 ng/mL. For lactate; a level of >2.5 mmol/L, found in 64.9% (96/148) of the sepsis patients, gave an optimized sensitivity and specificity (Table 2). In the control group, 14 patients (50.0%) had an elevated HBP level (>15 ng/mL) at enrollment (Figure 1A). Seven of these patients died. The 14 had been resuscitated after cardiac arrest or had received massive blood transfusions. Eight of them were subsequently diagnosed with infections during their stay in the ICU. Among the controls, lactate levels were elevated (>2.5 mmol/L) in 14 (56.0%) of the 25 available enrollment samples (Figure 1B).

**Table 2:** Laboratory measurements at enrollment

Characteristics	Sepsis group Number = 126 (%)	Severe sepsis Number = 25 (%)	Control group Number = 28 (%)	*P*-values
HBP ng/mL - n sampled	125	24	28	
HBP ≥15 ng/mL	91 (72.8)	18 (75.0)	14 (50.0)	<0.01
HBP/WBC ratio >2	78 (62.4)	8 (33.3)	12 (42.9)	
HBP ≥15 ng/mL and/or HBP ratio >2	110 (88.0)	19 (79.2)	14 (50.0)	
Lactate mmol/L	n = 123	n = 25	n = 25	0.39
Lactate >4	47 (38.2)	7 (28.0)	8 (32.0)	
Lactate 2.5- 3.9	39 (31.7)	6 (24.0)	6 (24.0)	
Lactate 0-2.49	37 (30.1)	12 (48.0)	11 (44.0)	
CRP ng/mL	n = 126	n = 25	n = 26	<0.001
CRP >200	90 (71.4)	14 (56.0)	1 (3.9)	
CRP 100 -199	22 (17.5)	5 (20.0)	2 (7.7)	
CRP 0-99	14 (11.1)	6 (24.0)	23 (88.5)	

CRP, C-reactive protein; HBP, heparin-binding protein; n, number.

In the whole study group (n = 179), both HBP and lactate levels at enrollment were significantly higher among 28 day non-survivors compared to survivors (*P *<.05). There were correlations between HBP concentration at enrollment and WBC (rho =.31), lactate (rho =.24), SOFA score (rho =.27), IL-6 (rho =.22), IL-8 (rho =.19), D-dimer (rho =.38), activated partial thromboplastin time (APTT) (rho =.25), creatinine (rho =.19) and bilirubin (rho =.28). Lactate levels at enrollment correlated to the same parameters and also to numbers of ICU days (rho =.22), APACHE II score (rho =.25), INR (rho =.29), ventilator days (n =.22) and platelet numbers (rho =.27). CRP only correlated with creatinine (rho =.25), IL-6 (rho =.31) and hospital days (rho =.19).

In patients with positive blood cultures, a total of nine different Gram-positive, eight different Gram-negative bacteria species, and one Candida species were found. All species were found with an elevated HBP level, except in one case of *Enterobacter cloacae *bacteremia.

No significant differences in HBP levels were found between Gram positive and Gram negative bacteremia (*P *=.93).

### HBP/WBC ratio and sepsis-induced transient leukopenia

Some patients with severe sepsis develop a transient leukopenia. Since HBP is released by neutrophils, the calculation of a HBP (ng/mL)/WBC (10^9^/L) ratio in cases of transient leukopenia was suggested in a previous study, in which a ratio of >2 indicated an increased risk of developing severe sepsis [20]. In the sepsis group, 26 of 149 (17.5%) patients had a transient leukopenia with an initial WBC count of <4 (10^9^/L), which was normalized during the study period of 144 hours. All of these 26 patients had a 'positive' HBP/WBC ratio of >2 (Figure 1F). Despite the low WBC count, 15 (61.5%) of them had an increased HBP level (≥15 ng/mL) (Figure 1E) and another six patients had an elevated HBP level at the second sampling after 24 hours. Six sepsis patients had a persistent leukopenia (<2 × 10^9^/L), due to chemotherapy for hematological malignancies. These patients had low HBP levels, but a HBP ratio >2 during the whole study period (Figure 1 E-F).

In the control group, four patients had a WBC count of <4 × 10^9^/L at inclusion but also a HBP level of ≥15 ng/mL. In this group, calculation of the HBP/WBC ratio did not increase the numbers of patients with a positive HBP result (Figure 1E, F).

### The combined use of HBP concentration and HBP/WBC ratio

The combined use of an elevated HBP concentration (≥15 ng/mL) and HBP ratio (>2) at enrollment identified 130 (87.2%) of the patients with sepsis. Of the 19 patients in the sepsis group who were HBP-negative at enrollment, another eight patients had an increased HBP level (≥15 ng/mL) in the 24 hour sample. Thus, 138 (92.6%) of the patients with severe sepsis/septic shock had an elevated HBP level (concentration and/or ratio) within 24 hours of enrollment in the ICU. As a group, the remaining 11 HBP-negative patients were somewhat less ill than the rest of the sepsis cohort: four patients had an elevated lactate level (>2.5 mmol/L), the mean SOFA score was 7 (compared to 10.1 in the whole sepsis group) and the mean APACHE II score was 19 (compared to 21.4) and only one died.

### Serial HBP measurements

**Figure 2:**
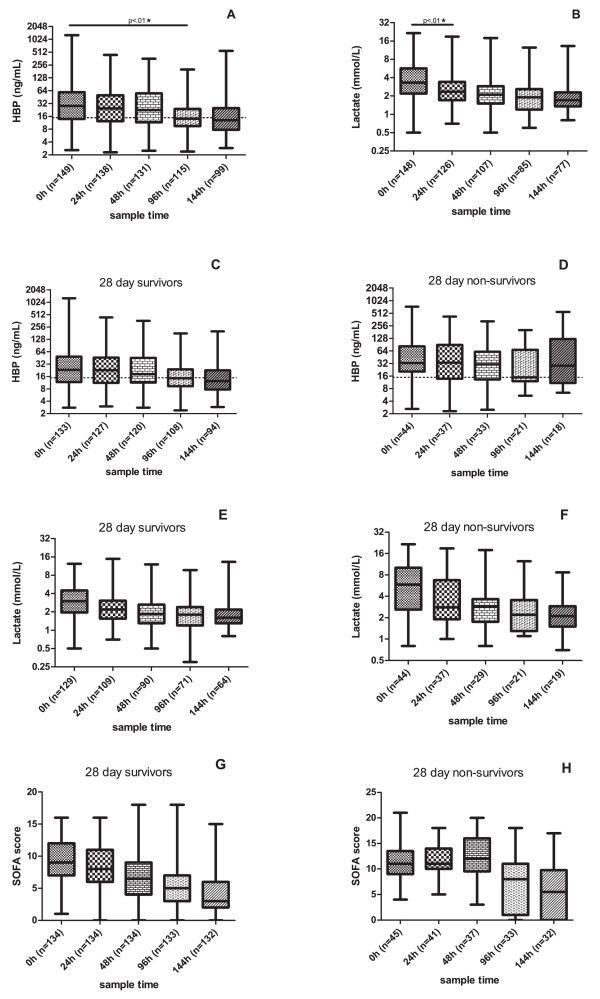
**HBP and lactate concentration over time and 28 day survival**. Results from consecutive samplings for HBP (**A**) and lactate (**B**) over the study period. Plasma concentration for HBP (**C-D**) and lactate (**E-F**) in survivors and non-survivors after 28 days. SOFA score at enrollment in survivors and non-survivors (**G-H**). Mann-Whitney´s non-parametric test was used for comparisons between the groups and *P *-values are given. Star symbols indicate a significant difference (*P *<.05). The suggested cut-off value for HBP is marked at 15 ng/mL. Median values are given. HBP, heparin-binding protein; SOFA, Sepsis-related Organ Failure Assessment.

Serial plasma concentrations were available for HBP, lactate and CRP. The median plasma concentrations of these three parameters (Figure 2 A, B) declined in the sepsis group over time during the study period. The median HBP levels were higher at each sampling time point among non-survivors compared to those who survived and increased in the group of patients who died within 28 days of enrollment (Figure 2C, D). In contrast, lactate and CRP (Figure 2E, F) did not show an increasing trend among non-survivors.

Comparing the last measured HBP and lactate concentrations between survivors and non-survivors, revealed that both HBP and lactate levels were significantly higher in 28 day non-survivors (*P *<.01).

### HBP and 28-days mortality

**Figure 3:**
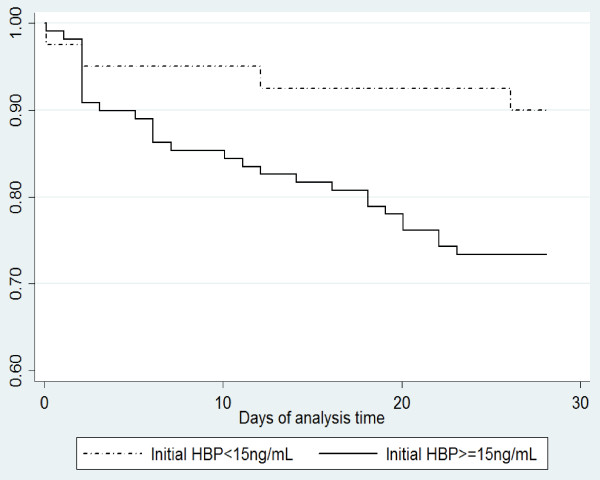
**Kaplan-Meier survival estimates of 28-days mortality according to HBP-level >15 ng/mL or <15 ng/mL at inclusion**. HBP, heparin-binding protein.

A total of 34 patients died within 28 days among the sepsis patients, 26 (20.6%) among those with septic shock and 8 (32.0%) among those with severe sepsis, corresponding to an overall case-fatality rate of 26.4% (95% confidence interval (95% CI): 18.8 to 36.9) per 28 days. The 28-day case-fatality rate was increased four-fold among sepsis patients with initial HBP >15 ng/mL, estimated by Cox regression, adjusted for leukopenia, s-bilirubin and s-creatinine levels (aHR:4.1 (95% CI:1.3 to 12.6)) (Figure 3). Although the stratified analyses were based on small numbers, the results were largely consistent in both sepsis groups and there was no evidence for interaction (*P *=.18).

### Controls that developed infection in the ICU

In the 14 control patients who developed clinical signs of an infection during their stay in the ICU, eight had a concomitant rise of HBP. One patient developed infection after transfer to another hospital and was only sampled once, thus precluding evaluation of changes in HBP. Five patients developed clinical infection with no corresponding rise of HBP. One of these had a Herpes Zoster infection with little other signs of inflammation. The remaining four were all admitted for cardiac arrest that was later complicated by aspiration pneumonia, fever and rising CRP. However, only one of the four was hemodynamically unstable at the time that pneumonia developed.

## Discussion

This is the first study investigating plasma concentrations of HBP in patients with severe sepsis and septic shock in the ICU. We found that admission and serial HBP measurements may be tools for predicting outcome in patients with severe sepsis or septic shock. Elevated HBP-levels at admission were associated with an increased case-fatality rate at 28 days. Serial SOFA score and lactate, which are established markers of outcome in critically ill patients, and HBP, were all significantly higher among 28-day non-survivors compared to survivors at enrollment and 24 hours, but of these three, only HBP was significantly elevated in non-survivors compared to survivors at 144 hours.

In all, we found elevated HBP levels in 87% of the septic patients at study enrollment, using a cut-off of 15 ng/mL and/or a HBP/WBC ratio of >2. This result is in line with a previous study in the emergency department, which suggested that plasma HBP is an early marker for severe sepsis and circulatory failure [20]. However, the studied cohorts differ in many ways: in contrast to the previous study on ED patients, samples in the present study were collected in the ICU, at a later time point in the course of disease and 80% of the patients were already on treatment with vasopressors, due to circulatory failure, at the time of first sampling. Many patients had serious underlying diseases, such as malignancies and chemotherapy-induced leukopenia. Also, the spectrum of infectious foci was different with a large proportion of patients having abdominal and post-operative infections.

Transient leukopenia, which was found in 17.6% of the patients, is a relatively common feature of sepsis [29]. Neutrophils are considered to be the main source of HBP and consequently, none of the patients with persistent leukopenia due to hematological malignancies had elevated HBP concentrations. Yet, 61.5% of the patients with transient leukopenia had high HBP levels, indicating that white blood cells, although temporarily decreased, are still present in the body and neutrophils are activated. Possible explanations for the low WBC seen in septic patients are: enhanced exocytosis, exhaustion or arrest of maturation of bone marrow cells, tissue secernation of neutrophils, or tethering to the endothelium during septic episodes [30,31]. Unsurprisingly, use of the HBP/WBC ratio increased sensitivity in diagnosing patients with transient leukopenia.

A large proportion of patients in the control group also presented with elevated HBP levels, which indicates that HBP, like all inflammatory biomarkers, is an acute phase reactant, not entirely specific for infections. It is worth noting that even though 28-day mortality was almost twice as high in controls, median HBP at inclusion was only half that of the septic patients. One could speculate that serious events such as cardiac arrest or massive blood transfusions lead to neutrophil activation and the subsequent release of HBP, but not to the same extent as in serious infections. Also, most of the patients with cardiac arrest developed early-onset pneumonia, probably as a result of aspiration, and thus a likely neutrophil activation due to both inflammatory and infectious insult. Although control patients were classified as non-infected at enrollment, 50% subsequently developed infections during the study period. Most of the controls that developed infections had a concomitant rise in HBP. Those that did not rise in HBP were, except for one patient, hemodynamically stable.

A total of 17 different bacterial species were cultured from blood with *Escherichia coli *and *Streptococcus pneumoniae *the most common findings. All but one of the bacterial species was found together with an elevated HBP concentration. This, together with the finding that HBP levels were significantly higher among patients with positive blood cultures, supports previous findings that most bacteria are capable of inducing HBP release [19].

The present study has several strengths. It was prospective with a relatively large sample size, involving a broad range of clinical presentations and diagnoses. The diagnoses and bacterial culture findings are in line with previous ICU studies and reflect a spectrum of patients that are likely to be encountered if HBP was used as a test in critically ill ICU patients. Inclusion criteria for infected patients were identical in the two patient cohorts and repeated blood samples were collected during the first six days after enrollment in a large majority of the study group.

Limitations of the study involve the control group, which was small in size and composition, thus restricting generalizability. An unexpected difficulty was that, even though the planned number of controls to include was much lower than septic patients, their recruitment took longer, despite wide inclusion criteria. A significant problem, when recruiting ostensibly non-infected ICU control patients, is that many will develop infections, as was the case in our study. This makes evaluation of HBP-levels difficult - are levels increased due to non-infectious causes or to an incipient infection? Due to these circumstances, we have restrained inferences from control patients to the day of study inclusion, at which time we believe they were truly non-infected or incipient infections were of minor relevance. Finally, the study was single center and we lack an extended comparison with some biomarkers, notably procalcitonin.

## Conclusions

In conclusion, this study on critically ill patients with severe sepsis/septic shock shows: that plasma HBP levels were significantly higher in patients with severe sepsis or septic shock compared to patients with non-septic illness in the ICU; that HBP was associated with severity of disease; that elevated HBP at admission was associated with an increased risk of death; and that HBP that rises over time may identify patients with a deteriorating prognosis. Previous findings on HBP as a sensitive marker for severe sepsis were confirmed in a different setting, underlining its potential as a valuable diagnostic tool.

## Key messages

• Plasma levels of the neutrophil-derived heparin-binding protein (HBP) were significantly higher among patients with severe sepsis and septic shock as compared to non-infected critically ill ICU patients.

• At admission to the ICU, an elevated HBP level was found in 87.2% out of 151 septic patients.

• An elevated initial HBP level was associated with an increased case-fatality rate at 28 days in septic patients.

• HBP, lactate levels and SOFA scores were all significantly higher at enrollment and 24 hours among 28-day non-survivors compared to survivors, but only HBP levels were significantly higher at 144 hours.

• The results indicate that admission and serial HBP measurements may be additional tools for predicting outcome in patients with severe infections.

## Abbreviations

APACHE: Acute Physiology and Chronic Health Evaluation; CI: confidence interval; CRP: C-reactive protein; ELISA: enzyme-linked immunosorbent assay; HBP: heparin-binding protein; IL: interleukin; SIRS: systemic inflammatory response syndrome; SOFA: Sepsis-related Organ Failure Assessment; WBC: white blood cells.

## Competing interests

Hansa Medical AB that partly funded this study, but had no influence on the study design, data analysis and writing of the manuscript, has filed a patent application on the use of HBP as a diagnostic tool in sepsis. AL^1 ^and PÅ are listed as inventors. The patent application is pending. Authors MI, CJT, AL^2 ^and JSC have no competing interests to declare.

## Authors' contributions

AL^1 ^performed the HBP analysis, analyzed data and wrote parts of the manuscript. PÅ analyzed data and wrote parts of the manuscript. MI performed statistical analysis and wrote parts of the manuscript. CJT participated in the study design and was responsible for including patients and data collection. AL^2 ^carried out IL-6 and IL-10 analyses and collected data. JSC participated in the study design, enrolled patients, collected and analyzed data and wrote parts of the manuscript. All authors read and approved the final manuscript.
